# Supplementary material for: Trends in prevalence of acute stroke impairments: A population-based cohort study using the South London Stroke Register
Source: PLoS Med. 2020 Oct 9;17(10):e1003366. doi: 10.1371/journal.pmed.1003366 (PMC7546484; doi:10.1371/journal.pmed.1003366)
Supplement: S1 Table — NIHSS, National Institutes of Health Stroke Scale. (DOCX) [file pmed.1003366.s002.docx]

**S1 Table.** Trends in the median NIHSS score over time, stratified by aetiological subtype of stroke

| **Stroke subtype** | **NIHSS score, median (IQR)** | | | | | | **P-value*** |
| --- | --- | --- | --- | --- | --- | --- | --- |
|  | **2001-2003** | **2004-2006** | **2007-2009** | **2010-2012** | **2013-2015** | **2016-2018** |  |
| **LAA** | 10.0 (5.8-15.0) | 5.0 (3.0-11.0) | 8.0 (4.0-15.0) | 7.0 (2.5-11.5) | 8.0 (3.0-12.2) | 5.5 (2.0-12.2) | 0.038 |
| **CE** | 10.0 (5.0-14.0) | 8.0 (4.0-17.0) | 11.0 (5.0-19.8) | 5.0 (2.8, 12.0) | 6.0 (3.0-16.5) | 6.0 (3.0-14.0) | <0.001 |
| **SVO** | 5.0 (3.0-7.0) | 4.0 (3.0-6.0) | 4.0 (3.0-6.0) | 3.0 (2.0-5.8) | 4.0 (3.0-6.0) | 4.0 (2.0-6.0) | 0.006 |
| **UND** | 7.0 (4.0-14.0) | 7.0 (4.0-12.0) | 7.0 (4.0-15.0) | 6.0 (3.0-12.8) | 5.5 (3.0-9.0) | 5.0 (2.0-10.0) | <0.001 |
| **PICH** | 14.0 (6.0-20.0) | 15.0 (7.0-25.0) | 14.0 (5.0-25.2) | 7.0 (3.8-15.5) | 7.0 (4.0-16.0) | 11.0 (4.2-17.2) | <0.001 |
| **SAH** | 1.0 (0.0-18.0) | 2.0 (0.0-15.0) | 18.0 (0.8-31.0) | 4.0 (1.0-20.0) | 1.0 (0.0-5.8) | 0.0 (0.0-3.0) | 0.106 |

*Chi-squared test for trend
