# Supplementary figures and images for: Trends in prevalence of acute stroke impairments: A population-based cohort study using the South London Stroke Register
Source: PLoS Med. 2020 Oct 9;17(10):e1003366. doi: 10.1371/journal.pmed.1003366 (PMC7546484; doi:10.1371/journal.pmed.1003366)

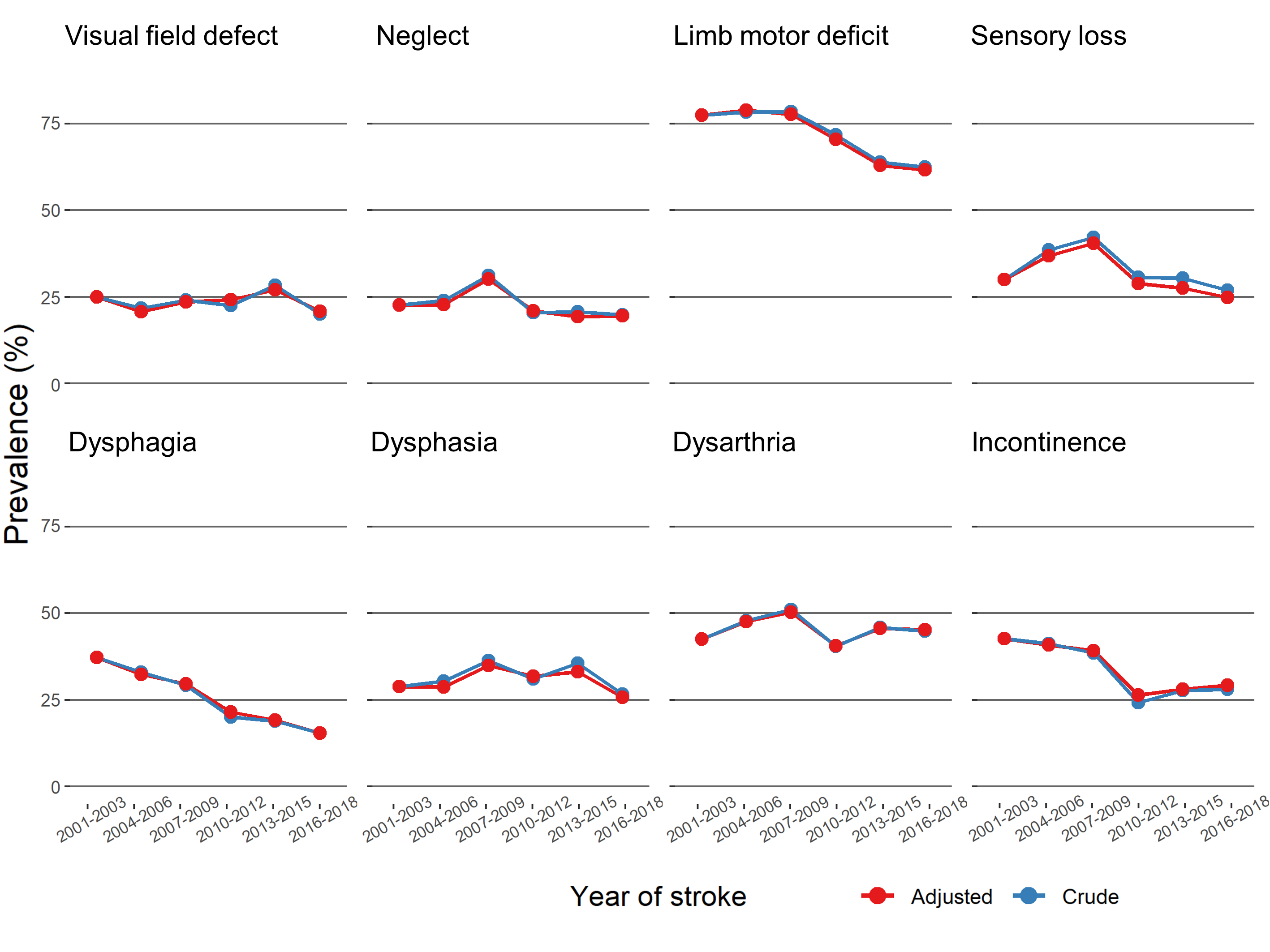

Supplement: S1 Fig — AF, atrial fibrillation; MI, myocardial infarction; TOAST, Trial of Org 10172 in Acute Stroke Treatment. (TIF) [file pmed.1003366.s003.tif]

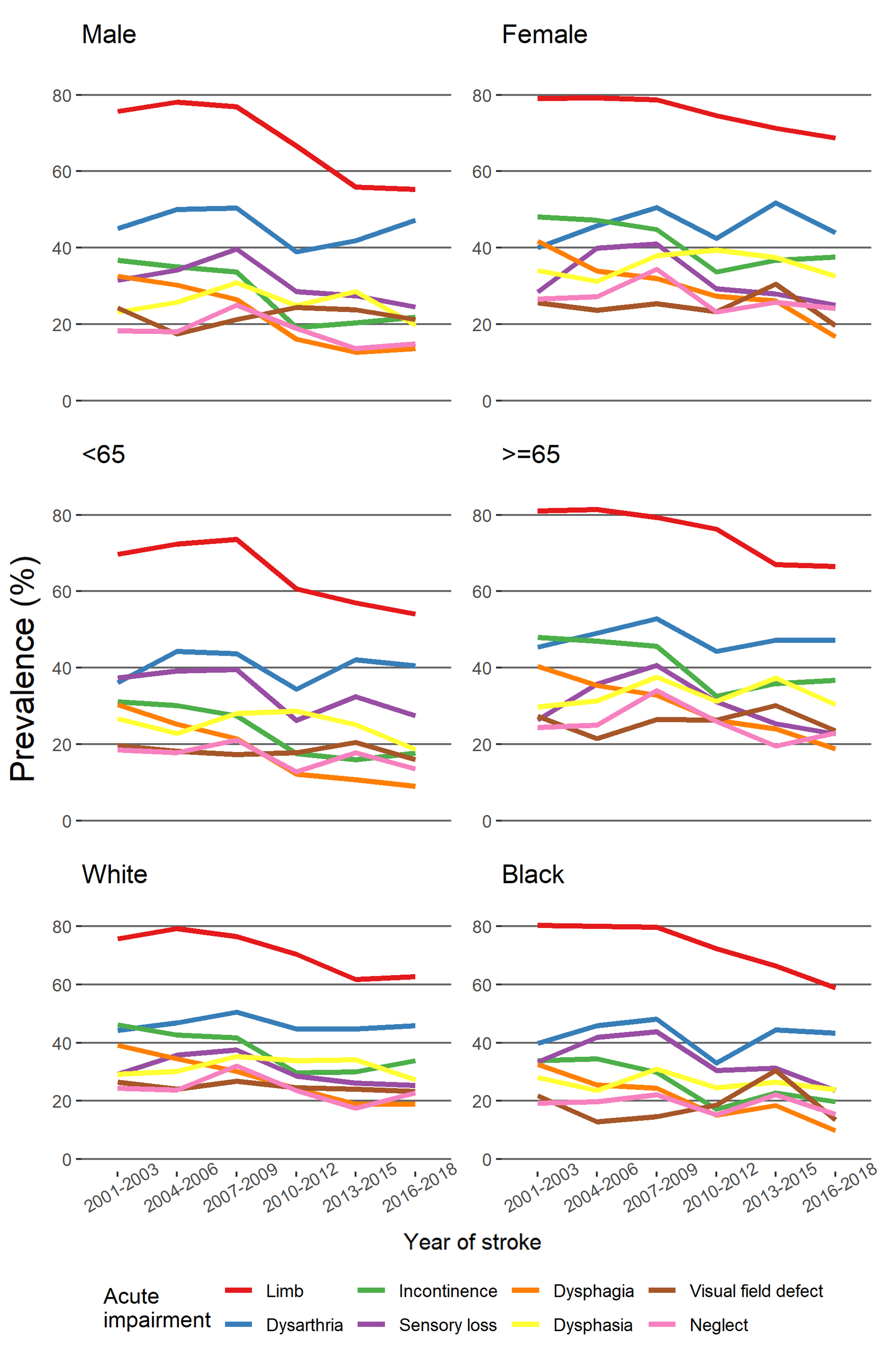

Supplement: S2 Fig — AF, atrial fibrillation; MI, myocardial infarction; TIA, transient ischaemic attack; TOAST, Trial of Org 10172 in Acute Stroke Treatment. (TIF) [file pmed.1003366.s004.tif]

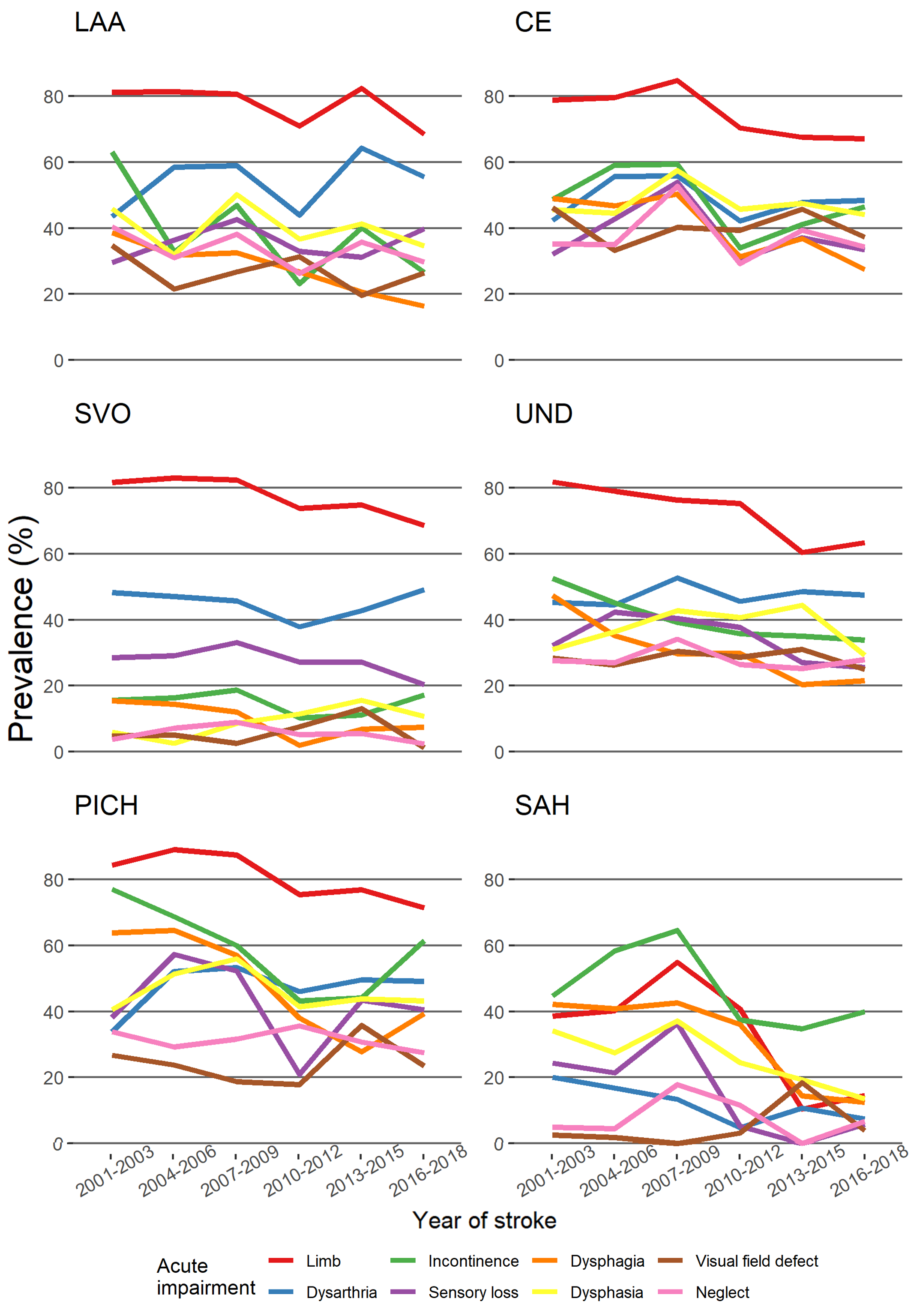

Supplement: S3 Fig — TOAST, Trial of Org 10172 in Acute Stroke Treatment. (TIF) [file pmed.1003366.s005.tif]
