# Supplementary material for: Trends in prevalence of acute stroke impairments: A population-based cohort study using the South London Stroke Register
Source: PLoS Med. 2020 Oct 9;17(10):e1003366. doi: 10.1371/journal.pmed.1003366 (PMC7546484; doi:10.1371/journal.pmed.1003366)
Supplement: Attachment — Submitted filename: PLOS_ReviewerComments_Response_2020-07-22.docx [file pmed.1003366.s006.docx]

Dear Dr. Clare Stone,

Many thanks for the valuable and constructive comments from the reviewers, which we hope we have addressed below to improve our manuscript.

**-----------------------------------------------------------**

**Requests from the editors:

*In the Abstract Methods and Findings section, please include a brief note summarising any key limitations of the study methods.**

*We have now included a sentence on limitations in the Methods and Findings section of the Abstract (pg. 2, lines 32-34).* ***At this stage, we ask that you include a short, non-technical Author Summary of your research to make findings accessible to a wide audience that includes both scientists and non-scientists. The Author Summary should immediately follow the Abstract in your revised manuscript. This text is subject to editorial change and should be distinct from the scientific abstract. Please see our author guidelines for more information:** [**https://eur03.safelinks.protection.outlook.com/?url=https%3A%2F%2Fjournals.plos.org%2Fplosmedicine%2Fs%2Frevising-your-manuscript%23loc-author-summary&amp;data=01%7C01%7Camanda.clery%40kcl.ac.uk%7Cb6ecf9493a7448cbb28f08d81e7eb39e%7C8370cf1416f34c16b83c724071654356%7C0&amp;sdata=CyAzWqOOxRd%2FCqqO577EV2peg9d6nxR3kdWL9dwcT8k%3D&amp;reserved=0**](https://eur03.safelinks.protection.outlook.com/?url=https%3A%2F%2Fjournals.plos.org%2Fplosmedicine%2Fs%2Frevising-your-manuscript%23loc-author-summary&amp;data=01%7C01%7Camanda.clery%40kcl.ac.uk%7Cb6ecf9493a7448cbb28f08d81e7eb39e%7C8370cf1416f34c16b83c724071654356%7C0&amp;sdata=CyAzWqOOxRd%2FCqqO577EV2peg9d6nxR3kdWL9dwcT8k%3D&amp;reserved=0)

*Please find the author summary now added following the abstract on pg. 4 (lines 39 to 59).* ***Please clarify in the paper if the analytical approach followed here corresponded to one laid out in a prospective study protocol or analysis plan? Please state this (either way) early in the Methods section.

a) If a prospective analysis plan (from your funding proposal, IRB or other ethics committee submission, study protocol, or other planning document written before analyzing the data) was used in designing the study, please include the relevant prospectively written document with your revised manuscript as a Supporting Information file to be published alongside your study, and cite it in the Methods section. A legend for this file should be included at the end of your manuscript.

b) If no such document exists, please make sure that the Methods section transparently describes when analyses were planned, and when/why any data-driven changes to analyses took place. 

c) In either case, changes in the analysis-- including those made in response to peer review comments-- should be identified as such in the Methods section of the paper, with rationale.**
*No specific funding was granted to conduct this particular study, therefore there is no funding proposal and accompanying analysis plan. However, we have now added clarity as to when the analysis was planned in the methods section (pg.7, lines 94-95).*

***We'd suggest using an appropriate reporting guideline (eg STROBE -** [**https://eur03.safelinks.protection.outlook.com/?url=https%3A%2F%2Fwww.equator-network.org%2Freporting-guidelines%2Fstrobe%2F&amp;data=01%7C01%7Camanda.clery%40kcl.ac.uk%7Cb6ecf9493a7448cbb28f08d81e7eb39e%7C8370cf1416f34c16b83c724071654356%7C0&amp;sdata=bqZ80GAOkSXlzs0NgKFYBA%2BXzCqP5zRRyVdqNMapPGE%3D&amp;reserved=0**](https://eur03.safelinks.protection.outlook.com/?url=https%3A%2F%2Fwww.equator-network.org%2Freporting-guidelines%2Fstrobe%2F&amp;data=01%7C01%7Camanda.clery%40kcl.ac.uk%7Cb6ecf9493a7448cbb28f08d81e7eb39e%7C8370cf1416f34c16b83c724071654356%7C0&amp;sdata=bqZ80GAOkSXlzs0NgKFYBA%2BXzCqP5zRRyVdqNMapPGE%3D&amp;reserved=0)**) - to guide reporting of study methods and findings; if doing this please ensure you upload the completed STROBE checklist as supporting information alongside the revised paper.**

*We have now used the STROBE guideline, providing additional information in the manuscript where relevant, and included the checklist in the Supporting Information.* ***At the moment the Discussion section doesn't explicitly include a clear Strengths and Limitations section; with respect to the latter this should include a summary of any specific biases inherent in the study methods which might have prevented the observed effects from closely approximating the "real" effects, and if so in what direction those biases might have have affected observed outcomes (eg towards null or away from null).**

*We have now restructured the discussion section to include a Limitations section (pg.25 line 349-pg.26 line 369).* **-----------------------------------------------------------

Comments from the reviewers:

Reviewer #1: I confine my remarks to statistical aspects of this paper. These were well done and I recommend publication

Peter Flom**
*Thank you, we value your positive remarks on the statistical analysis.*

**-----------------------------------------------------------

Reviewer #2: Clery et al. report the trends of acute stroke impairments overtime using population-based data. Limb motor deficits, dysphagia and incontinence declined between 2001 and 2018. Interestingly, the prevalence of all pre-stroke risk factors, except for vascular disease, increased over time. The authors hypothesize that the increased use of anticoagulants and statins could count, at least in part, for the decrease of acute impairments.**

**- This study analyzes ischemic stroke, ICH and SAH. These diseases are different in etiology, severity and prognosis. The article is mostly focused on ischemic stroke. The authors may want to consider concentrating in ischemic stroke solely.**

*Thank you for your comment. Including all subtypes of stroke maintains our goal to provide results for the breadth of impairments for all stroke patients. We have adjusted for stroke subtype in analyses as well as discussed the breakdown of the impairments by ischemic classification and haemorrhagic subtypes with similar focus on both and therefore have decided that the analysis is not restricted to solely ischemic subtypes.*

**- Can the authors provide data on gaze paresis and dysarthria?**

*While gaze paresis is a valuable impairment, we were unable to include this because these data were not available for the entire study period. Dysarthria is available in our data and we have now included it in our analysis, thank you for the suggestion.*

**- Why did the authors exclude the data from 1995 to 2001?**

*Thank you for your query. This was (1) to ensure fair comparisons and (2) to avoid repetition. (1) TOAST data were not readily collected in this early time period, and as an important subgroup for analysis and adjustment we decided to include only those years of the study for which these data were collected. (2) A previous paper written by Lawrence et al. in 2001, cited in this study, looked at a similar research question in the late 1990s and therefore we did not deem it necessary to report these data again.*

**- The decrease in the use of anti platelets may be related to new data showing that there is no benefit on its use for primary prevention**

*Thank you for this suggestion, we have now clarified this in the discussion (pg. 22 lines 283-287).*

**- The results are important for policy makers, but I believe these data are population specific and cannot be extrapolated. also, the decrease in stroke severity, and its association with the use of antithrombotics has already been shown.**

*Thank you for this comment, we have now included this in the limitations section of our discussion (pg. 26 lines 365-369). However, we still believe that these data are relevant to policy makers and clinicians, particularly because we present results within subgroups so they can be interpreted in different population groups.*

**- "We have found that strokes of undetermined aetiology have high levels of impairments comparable to that of LAAs and CEs." Stroke of undetermined source (aka ESUS) are mostly due to covert afib or carotid plaques < 50%. This may also explain the reported findings.**

*Thank you for your comment, we have now added this to our discussion of strokes of undetermined aetiology (pg. 25 lines 343-344).* **-----------------------------------------------------------**

**Reviewer #3: This paper describes the trends in prevalence of acute stroke impairments in a large stroke registry. The results are interesting, and the analysis is convincing. I have just a few minor questions and comments to Authors.
1) page 6, line 83. Please give values in mg, most of us readers do not use mmols.**

*Thank you, we have updated this now in our methods section (pg.7 line 107).*

**2) page 7 line 88. Despite relevant negative comments about (see for instance Stroke 36(4):902-4 · May 2005 "Time to burn Toast"), Toast classification is still widely used. However, I suggest Authors, if at all possible, to use OCSP classification, and also to look at Esus cases, which could have different level of impairment.**

*Thank you for your suggestion. We acknowledge the limitations of the TOAST classification that you have highlighted. However, for our study we consider the TOAST classification to be more clinically relevant as it provides the etiological mechanisms of stroke, which is particularly relevant for offering an understanding of the changes in the prevalence of acute impairments over time. The OCSP is also limited by the fact that it uses clinical symptoms and signs including acute impairments such as motor and sensory deficits, dysphasia, and visual field defect (Lancet 1991; 337:1521-26), which would be inappropriate for this study which is analysing these impairments specifically.*

**3) page 7 line 92. As for haemorrhages, could Authors differentiate between typical and atypical forms? It seems that the latter are increased in recent years, and the impact on impairment may well be different.**

*Thank you for your comment and we agree that lobar and non-lobar haemorrhages may well impact acute stroke impairments however we unfortunately do not have this data available to us. We believe that our differentiation of primary intracerebral haemorrhage and subarachnoid haemorrhage provide a good understanding of acute impairments in haemorrhagic subtypes.*

**4) page 8 line 112. Authors did not adjust for smoking and cholesterol levels. Is there a specific reason for that? If not, could they add these two factors to their analysis?**

*Thank you for the suggestion. We have now updated our analyses to adjust for both cholesterol levels and smoking status throughout.*

**5) Results line 134. Mean age looks quite low, as compared to the one found in different European studies. Do Authors think that this fact could limit the external validity of their results?**

*Thank you for this important observation. Our cohort includes a high proportion of Black patients, typical of inner-city populations, who have a mean age of 64, as presented in the results. Therefore, this pulls down the mean age of the cohort compared to other European populations that are predominantly White. However, the mean age of our White population (71.7 years old) is comparable to the European average of 71 years old. By presenting the results by subgroup of age (<65 and 65 and over), we are able to capture the differences between age groups and ensures comparability to populations with an older average age.*

**6) Results line 152. The decrease in severity is evident. Hower, could it be due to more mild cases being admitted, or to less severe cases being admitted? is any other facility available for stroke patient in the study area? Have Authors any information on the number of patients staying at home with their stroke?**

*Thank you for your comment. Please see previously published research conducted by our group on this topic (Stroke 2016; 47:2269-2277). They identified that patients admitted to hospital had more severe strokes compared to those not admitted to hospital. But admissions to hospital have also increased over time, meaning that more milder strokes are likely detected, which we have acknowledged in the discussion. Our results also show however, that no one subtype of stroke has increased or decreased over time, suggesting there has not been a surge in the recognition of mild strokes.*

-----------------------------------------------------------

Many thanks again for the suggestions and we look forward to hearing from you soon.

Best wishes,

Amanda Clery
