# Supplementary material for: Trends in prevalence of acute stroke impairments: A population-based cohort study using the South London Stroke Register
Source: PLoS Med. 2020 Oct 9;17(10):e1003366. doi: 10.1371/journal.pmed.1003366 (PMC7546484; doi:10.1371/journal.pmed.1003366)
Supplement: Attachment — Submitted filename: PLOS_ReviewerComments_Response_2020-08-18.docx [file pmed.1003366.s007.docx]

Dear Dr. Clare Stone,

Many thanks for helpful comments from the editors. We hope we have addressed these below to improve our manuscript.

**-----------------------------------------------------------**

**Requests from Editors:**

**Competing interests: Please mention the YW is a paid statistical referee for PLOS Medicine, but had no role in the peer review of this paper.**

*We have now included this in the submission form.*

**Title – I suggest removing ‘The’**

*Thank you for the suggestion, we agree and have now updated (pg. 1 line 1).*

**Abstract – Please include summary demographic information and provide p values for 95% Cis (here and elsewhere). Also, Please quote one further limitation in the abstract**

*We have now added demographic information, 95% confidence intervals and p-values to the abstract (pg. 2 lines 24-28). We have also added an additional limitation (pg. 3 lines 37-38).*

**Author Summary – briefly please say what the 5 conditions are that were unaffected.**

*Thank you, these have now been added (pg. 4 line 55).*

**Please include a space before the opening square brackets for refs.**

*We have now added these spaces to references throughout the paper.*

**STROBE – please submit supp files individually, including this one and also sections and paragraphs should be used instead of pages as these change during formatting and revisions.**

*All supplementary files have now been uploaded individually. STROBE checklist has also been updated to reflect this comment.*

**- at line 35, suggest "In this study, we found that stroke patients in the SLSR had a complexity ..." (also similar amendments at line 56)**

*We have now updated these sentenced in the manuscript as recommended (pg. 3 line 39, and pg.4 line 60).*

**- Poor punctuation at line 312; in fact, this whole paragraph on ethnic disparities is quite hard to understand. Do you mean that the proportion of Black people in the relevant population has not changed, and therefore that a higher proportion of Black people than White have risk factors and suffer strokes in South London? Please clarify.**

*Thank you, we have amended this paragraph and hope that this has been clarified. We suggest there has been an increase in stroke incidence in the Black population over time, which may be due to the increase in risk factors know to affect the Black population more than White (pg. 19 lines 305-315).*

**Comments from Reviewers:**

**Reviewer #2: My concerns have been appropriately addressed.**

**Thank you for the opportunity of reviewing your paper.**

**Reviewer #3: Authors were kind enough to consider all my points, and, as far as possible, to follow my suggestions. I have no further question or comment.**

**-----------------------------------------------------------**

Many thanks and we look forward to hearing from you soon.

Best wishes,

Amanda Clery
